# Educational health disparities in hypertension and diabetes mellitus among African descent populations in the Caribbean and the USA: a comparative analysis from the Spanish town cohort (Jamaica) and the Jackson heart study (USA)

**DOI:** 10.1186/s12939-017-0527-9

**Published:** 2017-02-14

**Authors:** Aurelian Bidulescu, Trevor S. Ferguson, Ian Hambleton, Novie Younger-Coleman, Damian Francis, Nadia Bennett, Michael Griswold, Ervin Fox, Marlene MacLeish, Rainford Wilks, E. Nigel Harris, Louis W. Sullivan

**Affiliations:** 1 Department of Epidemiology and Biostatistics, Indiana University School of Public Health – Bloomington, Bloomington, IN USA; 2 Epidemiology Research Unit, Tropical Medicine Research Institute, The University of the West Indies, Kingston, West Indies Jamaica; 3 Chronic Disease Research Centre, Tropical Medicine Research Institute, The University of the West Indies, Bridgetown, West Indies Barbados; 4 Center of Biostatistics and Bioinformatics, University of Mississippi Medical Center, Jackson, MS USA; 5 University of Mississippi Medical Center, Jackson, MS USA; 6 Department of Medical Education, Morehouse School of Medicine, Atlanta, Georgia; 7 The University of the West Indies, Kingston, West Indies Jamaica; 8 The Sullivan Alliance, Alexandria, VA USA

**Keywords:** Hypertension, Diabetes, Education, Health disparities, Social determinants, Jamaica, Jackson Heart Study, Blacks, African Americans, Afro-Caribbean

## Abstract

**Background:**

Studies have suggested that social inequalities in chronic disease outcomes differ between industrialized and developing countries, but few have directly compared these effects. We explored inequalities in hypertension and diabetes prevalence between African-descent populations with different levels of educational attainment in Jamaica and in the United States of America (USA), comparing disparities within each location, and between countries.

**Methods:**

We analyzed baseline data from the Jackson Heart Study (JHS) in the USA and Spanish Town Cohort (STC) in Jamaica. Participants reported their highest level of educational attainment, which was categorized as ‘less than high school’ (<HS), high school (HS) and ‘more than high school’ (>HS). Educational disparities in the prevalence of hypertension and diabetes were examined using prevalence ratios (PR), controlling for age, sex and body mass index (BMI).

**Results:**

Analyses included 7248 participants, 2382 from STC and 4866 from JHS, with mean age of 47 and 54 years, respectively (*p* < 0.001). Prevalence for both hypertension and diabetes was significantly higher in the JHS compared to STC, 62% vs. 25% (*p* < 0.001) and 18% vs. 13% (*p* < 0.001), respectively. In bivariate analyses there were significant disparities by education level for both hypertension and diabetes in both studies; however, after accounting for confounding or interaction by age, sex and BMI these effects were attenuated. For hypertension, after adjusting for age and BMI, a significant education disparity was found only for women in JHS, with PR of 1.10 (95% CI 1.04–1.16) for < HS vs > HS and 1.07 (95% CI 1.01–1.13) for HS vs > HS. For diabetes; when considering age-group and sex specific estimates adjusted for BMI, among men: significant associations were seen only in the 45–59 years age-group in JHS with PR 1.84 (95% CI 1.16–2.91) for < HS vs > HS. Among women, significant PR comparing < HS to > HS was seen for all three age-groups for JHS, but not in STC; PR were 3.95 (95% CI 1.94–8.05), 1.53 (95% CI 1.10–2.11) and 1.32 (95% CI 1.06–1.64) for 25–44, 45–59 and 60–74 age-groups, respectively.

**Conclusion:**

In Jamaica, educational disparities were largely explained by age, sex and BMI, while in the USA these disparities were larger and persisted after accounting these variables.

**Electronic supplementary material:**

The online version of this article (doi:10.1186/s12939-017-0527-9) contains supplementary material, which is available to authorized users.

## Background

African-origin individuals in the United States have a higher prevalence of hypertension (HTN) and diabetes mellitus (DM) compared to Caucasians [1]. Several health disparities are related to these indicators, with the difference in education between African-descent individuals and Caucasians being one of the most important [2, 3]. In addition, the current obesity epidemic affects ethnicities differently, with African-descent individuals in the U.S. having consistently higher obesity rates than all other racial and ethnic groups resident in the U.S. [4]. Body mass index (BMI) increased steadily in all race-sex and education groups from 1997 to 2008, and African Americans (particularly women) had a consistently higher BMI than their white counterparts [5]. Important to note, overweight/obesity trends and racial disparities had larger increases over time among individuals with higher education levels, compared to their counterparts with lower education levels [5].

There are important educational differences between African descent populations in the Caribbean and in the United States [6, 7]. As those educational disparities might partly explain the differences in the prevalence of hypertension and diabetes mellitus between the two African-descent populations, we conducted a study with the following overall aim. Acknowledging that few studies have explored among African-descent individuals the prevalence of hypertension and diabetes according to levels of educational attainment the aim was to evaluate whether there are significant differences in estimates of educational health disparity for hypertension and diabetes mellitus comparing Afro-Caribbean individuals in Jamaica and African Americans in the United States. Specifically we aimed to estimate the age adjusted prevalence, prevalence ratios and prevalence differences for hypertension and diabetes mellitus by education categories for participants in the Spanish Town Cohort Study and the Jackson Heart Study and determine whether the patterns of disparity for the education differ between the two studies. Such analyses should add insights into the different stages of epidemiological transition (i.e., change in the disease pattern) that African-descent individuals in both settings are tangled in, and add information to the much debated ‘thrifty gene hypothesis’ that has so many applications to cardiometabolic diseases such as hypertension and diabetes.

## Methods

### Participants and source cohorts

The Jackson Heart Study (JHS) is an all African-American cohort study which enrolled 5306 participants between September 2000 and March 2004 [8, 9]. Participants were enrolled from three counties in the Jackson Mississippi metropolitan area, with the primary sample being between 35 and 84 years old and an additional sub-sample of 1499 members from the family of the primary sample members, who were 21 years old or older [8]. Details of the study design, recruitment process, and sample characteristics have been previously published [8–10]. The study was approved by the Institutional review boards of Jackson State University, Tougaloo College and the University of Mississippi Medical Center. In order to match the age range of the Spanish Town Cohort, for this study we included 4866 participants who were 25–74 years old who had complete data on age, educational attainment, hypertension prevalence and diabetes prevalence. Among excluded participants 19 had missing information for education and 52 for hypertension.

The Spanish Town Cohort Study (STC) was initiated in 1993 as part of an international study of hypertension, diabetes and chronic disease in African origin populations. Details of the study design, methods and procedures have been previously published [11–13]. Participants were 2654 individuals between ages 25–74 years and were recruited by door to door solicitation of eligible residents residing within randomly selected enumeration districts in Spanish Town, an urban community, located 15 miles from the capital city of Kingston, in Jamaica. The baseline cohort included 2654 persons who were evaluated between 1993 and 2001. The protocol for the study was reviewed and approved by the Ethics Committee of the Faculty of Medical Sciences, University of the West Indies, Mona. For this study we included 2382 participants with complete data on age, hypertension, diabetes and educational attainment. Among excluded participants 14 had missing information for education, 26 for hypertension and 172 for diabetes.

### Measurements and variable definitions/compositions

Data on demographic characteristics and educational attainment were collected via questionnaires in both studies. Educational attainment was collected based on questionnaire items on the highest level of completed education. In order to facilitate comparison across study sites the original education variables were collapse into three categories, namely: ‘less than high school’, high school and ‘more than high school’. The ‘more than high school’ category included persons who had vocational training, those who attended colleges and those who completed university degrees. The high school category included the same age range in Jamaica and in the USA.

Anthropometric and blood measurements for both studies were performed using standardized procedures [8, 11, 12]. Blood pressure measurement in the STC was performed using a mercury sphygmomanometer, with three measurements taken after the participants was seated for 5 min. The mean of the second and third measurements was used in the analysis. In the JHS, blood pressure was measured using a random-zero sphygmomanometer after the participant had been seated for 5 min; two readings were taken and the mean of the two used in the analysis [8, 9]. For both studies hypertension was defined as having a systolic ≥140 mmHg or diastolic blood pressure ≥90 mmHg or if participant reported being on medication for hypertension as recommended by the Seventh Report of the Joint National Committee on Prevention, Detection, Evaluation, and Treatment of High Blood Pressure [14].

Blood glucose was measured on a venous blood sample after an overnight fast in both studies [8, 13]. Diabetes mellitus was defined as a fasting blood glucose of 7.0 mmol/l (126 g/dL) or greater or if participants were on medication for diabetes, in accordance with the diagnostic criteria recommended by the American Diabetes Association [15].

All the other variables such as age, height, weight and the derived body mass index were assessed similarly within the two study cohorts.

### Statistical analyses

Analyses were performed using Stata 12.1 statistical software (Stata Corp., College Station Texas), and SAS 9.4 statistical software (SAS Institute Inc., Cary, North Carolina). We obtained crude and sex-specific estimates of the prevalence for hypertension and diabetes mellitus for each study and then category-specific estimates within education and age categories. Age-adjusted prevalence estimates, prevalence differences and prevalence ratios were obtained using Poisson regression models. We opted to use prevalence ratios as a measure of effect in this study because it has been shown to be a better measure of effect in studies where the prevalence of the outcome is high [16, 17]. We created separate models for diabetes and hypertension to derive adjusted estimates. Sex and age-group interactions were tested in the regression models and included in the final models if statistically significant. There was evidence for sex interaction in the relationship between education and both diabetes mellitus and hypertension, thus necessitating sex specific models. Additionally, there was age interaction for diabetes mellitus, therefore we presented age and sex-specific estimates for this outcome. BMI was included in all the models as a continuous variable. Age-adjusted and age-specific estimates were derived from models which included the interaction terms. We also assessed whether there was evidence for interaction by study site in order to assess whether there were differences in the patterns of education disparity in the JHS compared to the STC.

## Results

**Fig. 1:**
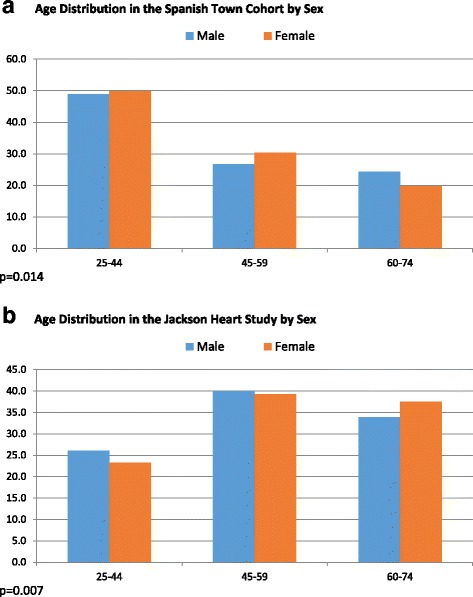
Age Distribution in the (**a**) Spanish Town Cohort Study and in the (**b**) Jackson Heart Study by Sex

Our analyses included 7248 participants, 2382 from the Spanish Town Cohort (STC) and 4866 from the Jackson Heart Study (JHS). Characteristics of participants by study site and sex are shown in Table 1. JHS participants were on average older, taller and heavier than their STC counterparts. Age distribution for participants at the two study sites are shown in Fig. 1a and b. Mean BMI was 31.2 kg/m^2^ in JHS vs 26.6 kg/m^2^ in the STC. JHS participants also had higher mean systolic and diastolic blood pressure and higher prevalence of hypertension (62% vs. 25%). Mean fasting blood sugar was similar in the two groups but the JHS participants had a higher prevalence of diabetes mellitus (18.2% vs. 12.5%). With regards to educational attainment there were significant differences between the two groups with 63% of JHS participants attaining post-secondary education compared to only 14% of STC participants. Findings were similar when sex-specific comparisons were performed (see Additional file 1: Table S1). As expected, there was evidence for a statistically significant association between age-group and educational attainment, as well as between age-groups and prevalent hypertension or diabetes mellitus (see Additional file 1: Figures S1A and S1B and S2B and S2B in the Appendix). There were significant sex differences for most of the characteristics studied (Additional file 1: Table S2) and evidence for interaction by sex in the relationship between education and both hypertension and diabetes mellitus, therefore we present sex-specific analyses for the rest of this manuscript.

**Table 1:** Characteristics of Study Participants in Spanish Town Cohort Study and Jackson Heart Study

Characteristic	Spanish Town Cohort	Jackson Heart Study	*p*-value
	*N* = 2382	*N* = 4866	
	Mean (SD)	Mean (SD)	
Age (years)	46.5 (13.7)	53.8 (11.3)	<0.001
Height (cm)	165.5 (8.7)	169.1 (8.8)	<0.001
Weight (kg)	72.6 (16.0)	91.2 (21.0)	<0.001
Body Mass Index (kg/m^2^)	26.6 (6.0)	31.9 (7.2)	<0.001
Systolic Blood Pressure (mmHg)	120.7 (21.1)	126.6 (17.3)	<0.001
Diastolic Blood Pressure (mmHg)	69.5 (13.9)	79.2 (10.1)	<0.001
Fasting Plasma Glucose (mmol/l)	5.53 (2.28)	5.52 (1.83)	0.914
	% (n)	% (n)	
Sex (male)	39.6 (944)	36.7 (1787)	0.016
Hypertension	25.0 (595)	62.2 (3025)	<0.001
Diabetes	12.5 (297)	18.2 (886)	<0.001
Education Category			<0.001
*Less than High School*	61.7 (1470)	16.4 (796)	
*High School*	24.1 (574)	20.3 (986)	
*More than High School*	14.2 (338)	63.4 (3084)

Table 2 shows the unadjusted prevalence of hypertension and diabetes mellitus for education categories within sex and study groups. Except for men in the STC, there was statistically significant differences (*p* < 0.001) in the prevalence of hypertension and diabetes mellitus by education level for all groups. For participants in the STC the prevalence of both diabetes and hypertension was lowest in the middle education group (i.e. those attaining high school level education). Among men, rates were similar in the ‘high school or more’ and ‘less than high school’ groups, while among women, prevalence was highest in the ‘less than high school’ group for both diabetes and hypertension. For participants in the JHS prevalence was generally lowest in the ‘more than high school’ group and highest in the ‘less that high school’ group.

**Table 2:** Prevalence of Hypertension and Diabetes Mellitus by Educational Categories for Spanish Town Cohort and Jackson Heart Study

Characteristic	Spanish Town Cohort		Jackson Heart Study	
	Male (*n* = 944)	Female (*n* = 1438)	Male (*n* = 1787)	Female (*n* = 3079)
Hypertension^1^				
*High School or More*	26.4 (33)	18.3 (39)	55.9 (634)	57.5 (1121)
*High School*	10.6 (25)	11.8 (40)	60.2 (212)	69.1 (438)
*Less than High School*	26.2 (153)	34.4 (305)	72.1 (217)	81.4 (403)
Diabetes^2^				
*High School or More*	10.4 (13)	11.3 (24)	18.8 (156)	16.2 (315)
*High School*	6.8 (16)	6.5 (22)	18.2 (64)	18.6 (118)
*Less than High School*	11.8 (69)	17.3 (153)	24.3 (73)	32.3 (160)

^1^Statistically significant differences (*p* < 0.001) in the prevalence of hypertension across education categories for both males and females in both studies. *P*-values for JHS accounts for clustering of persons within families

^2^Statistically significant differences (*p* < 0.001) in the prevalence of diabetes across education categories for females in both studies and males in JHS. For males in STC *p* = 0.088. *P*-values for JHS accounts for clustering of persons within families

Adjusted prevalence estimates for hypertension are shown in Table 3. Models were adjusted for age and BMI. Disparity patterns varied with both study and sex with evidence for interaction by sex and study site. Among men in the STC, age-adjusted prevalence for hypertension was highest among those with more than high school education and lowest among those with high school education (27% vs. 20%), while among women age adjusted prevalence of hypertension was highest among participants with less than high school education and lowest for those with high school education (28% vs 23%). In the JHS age adjusted prevalence of hypertension was highest in the less than high school education category and lowest in those with more than high school education for both men 62% vs 58%) and women (68% vs 62%). Statistically significant associations were seen only for women in JHS (*p* = 0.001 for higher prevalence of hypertension among lower education groups, assuming a linear trend).

**Table 3:** Sex-Specific Age-Adjusted Prevalence^1^ for Hypertension by Educational Categories

Characteristic	Spanish Town Cohort		Jackson Heart Study	
	Male	Female	Male	Female
Age-Adjusted Prevalence	%	%	%	%
*More than High School*	27.1 (19.9, 34.4)	25.4 (19.2, 31.5)	58.4 (55.6, 61.2)	61.6 (59.6, 63.7)
*High School*	20.0 (12.5, 27.4)	23.1 (16.2, 30.0)	60.3 (55.5, 65.1)	65.8* (62.7, 69.2)
*Less than High School*	21.7 (18.8–24.6)	27.5 (25.0, 30.0)	62.2 (57.5, 66.9)	67.6** (64.5, 70.7)

^1^Estimate derived from study and sex-specific Poisson regression models adjusted for age (entered as a categorical variable with three categories) and body mass index. Estimates take into account the clustering of persons within families for the JHS

*p < 0.05; **p < 0.01; ***p < 0.001, as based on prevalence differences

**Fig. 2:**
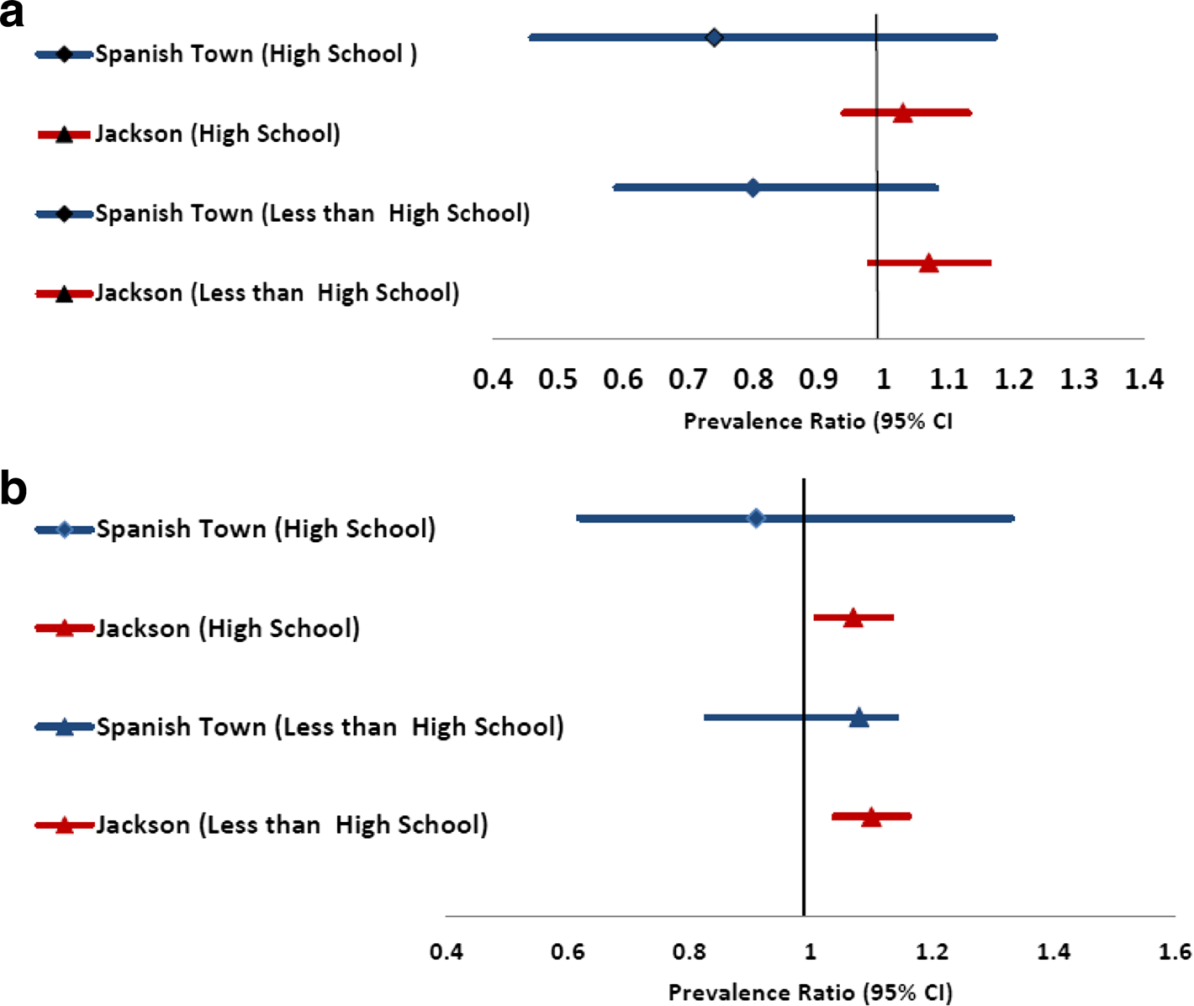
Sex and Age-Specific Prevalence^1^ for Hypertension by Educational Categories. Among Men (**a**) and among Women (**b**)

Results for diabetes are shown in Tables 4a and b. Again, there was some evidence for interaction by study site (*p* = 0.052 at education level three among women). Additionally there was interaction by age in the relationship between education level and diabetes prevalence for some age-groups (Fig. 1); we therefore present age and sex-specific estimates for each study site. Estimates were also adjusted for BMI. Among men in the STC, prevalence of diabetes was highest among those with high school education in the 45–59 years and the 60–74 years age-groups, but was lowest among men with high school education in the 25–44 years age-group (Fig. 2). Differences were however not statistically significant. For the JHS, there was a statistically significant higher prevalence of diabetes among men with less than high school education in the 45–59 years age-group with diabetes prevalence of 24% compared to 13% among those with more than high school education None of the other differences in prevalence across education categories were statistically significant. Among women in the STC, there was no clear pattern of association and none of the comparisons showed statistically significant differences. On the other hand, among women in the JHS there was a clear pattern of higher prevalence of diabetes among those with less than high school education in all three age-groups. This effect was largest among those in the 25–44 years age-group with diabetes prevalence of 23% among those with less than high school education compared to 6% among those with more than high school education.

**Table 4:** Sex and Age-Specific Prevalence1 for Diabetes Mellitus by Educational Categories

Characteristic	Spanish Town Cohort			Jackson Heart Study		
	25–44 years	45–59 years	60–74 years	25–44 years	45–59 years	60–74 years
Men						
*High School or More*	4.9 (0.1, 9.6)	10.1 (0.8, 19.3)	25.1 (5.9, 44.3)	7.1 (4.5, 9.6)	13.3 (10.3, 16.4)	23.0 (18.0, 28.1)
*High School*	1.9 (0.1, 3.8)	26.0 (9.6, 42.3)	45.9 (9.4, 82.4)	6.1 (1.0, 11.1)	18.9 (12.5, 25.3)	27.8 (19.7, 36.0)
*Less than High School*	5.3 (1.9, 8.6)	11.3 (7.1, 15.6)	19.9 (14.0, 25.9)	5.1 (0, 14.8)	24.4* (14.7, 34.2)	28.1 (21.7, 34.4)
Women						
*High School or More*	5.2 (1.8, 8.7)	16.3 (4.5, 28.1)	44.8 (25.2, 64.4)	5.7 (3.9, 7.6)	18.5 (16.0, 21.0)	25.4 (21.4, 29.4)
*High School*	5.8 (3.1, 8.4)	11.5 (1.0, 21.9)	14.4 (0, 41.2)	4.8 (1.1, 8.5)	16.5 (11.8, 21.3)	26.2 (20.9, 31.5)
*Less than High School*	5.3 (2.6, 8.0)	17.4 (13.6, 21.2)	30.1 (24.2, 36.0)	22.7* (8.2, 37.1)	28.2* (19.8, 36.5)	33.5* (28.6, 38.4)

*P < 0.05, as based on prevalence differences

^1^Estimate derived from study and sex-specific Poisson regression models account for interaction between age group and education category and adjusted for body mass index. Age group specific estimates obtained from post-estimation commands that used model derived coefficients to calculate adjusted prevalence estimates

Estimates take into account the clustering of persons within families for the JHS

**Fig. 3:**
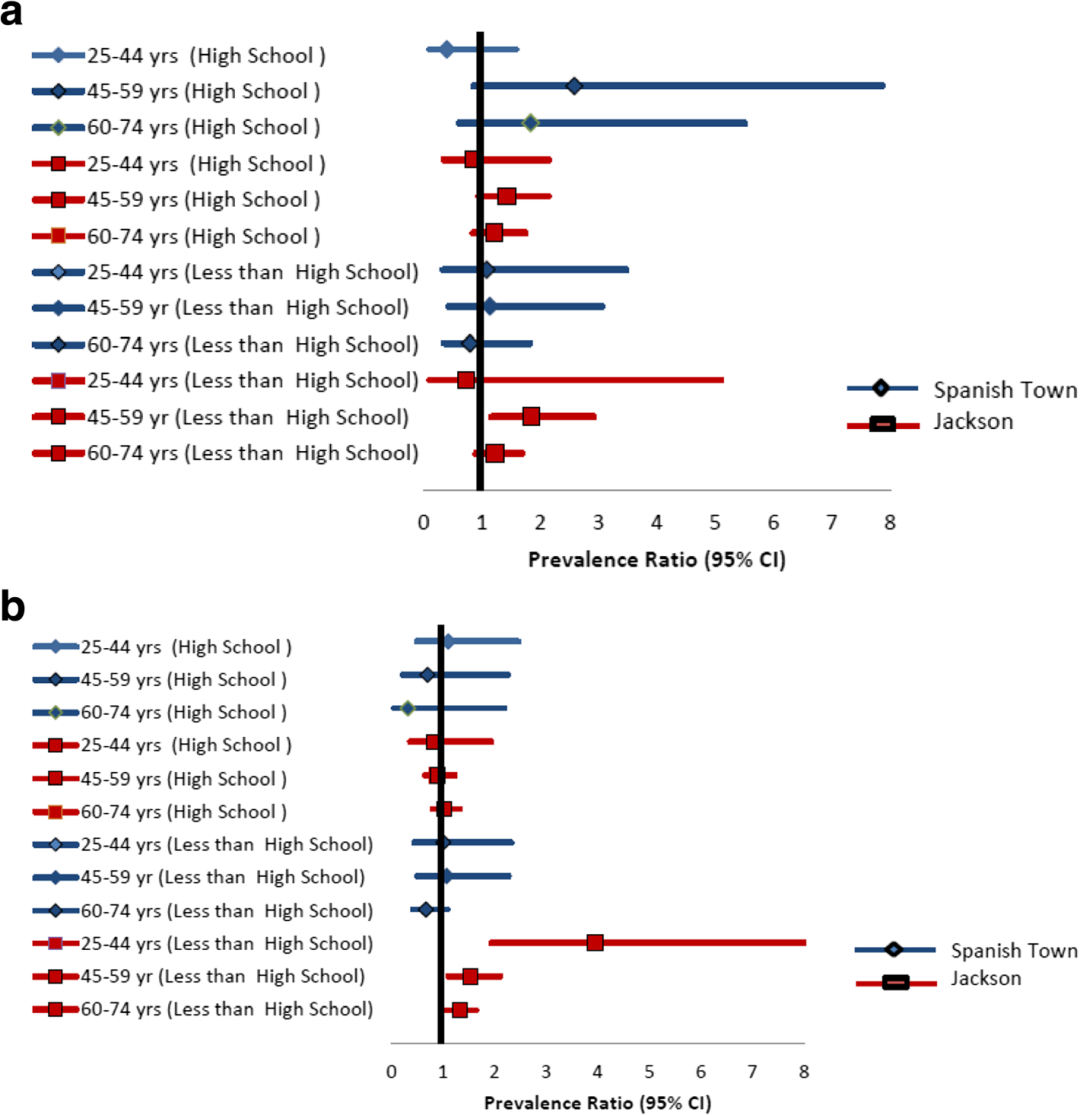
Sex and Age-Specific Prevalence^1^ for Diabetes Mellitus by Educational Categories. Among Men (**a**) and among Women (**b**)

Sex and study specific prevalence ratios and prevalence differences for hypertension with adjustment for age and BMI are shown in Fig. 3. Significant disparities were seen only for women in JHS. For diabetes, were present age-group specific estimated by study and sex groups with adjustment for BMI. Among men, significant disparities were seen only in the 45–59 age-group in the JHS while among women significant disparities were seen for all age-groups in JHS but not in STC.

## Discussion

Our study shows that there are differences in the patterns of educational health disparity for hypertension and diabetes mellitus when comparing African descent individuals in Jamaica and the USA. Much of the educational disparities seen in the Jamaican setting are explained by age sex and BMI such the differences become attenuated and no longer statistically significant. In the USA on the other hand much of the educational disparities persist after adjusting for these variables particular among women. Additionally there are differences in the patterns of disparity by study site and sex demonstrated in tests for interaction. Age group also had a significant effect on disparity patterns for diabetes. Men in the lower education groups had lower relative risk for hypertension in Jamaica but higher relative risk in the USA, but estimates did not achieve statistical significance. Among U.S. participants, there was a statistically significant higher prevalence of diabetes among men with less than high school education in the 45–59 years age-group compared with those with more than high school education. Among American women there was a clear pattern of higher prevalence of diabetes among those with less than high school education regardless of age-group.

Several health disparities are related to a higher prevalence of both hypertension and diabetes mellitus when comparing African descent individuals with Caucasians, the difference in education being one of the most important [2, 3]. Very few studies have assessed the relationship between education and outcomes such as hypertension or diabetes. Education was an important risk factor for the prevalence of hypertension and elevated blood pressure in a study of Chinese men and women [18]. Education appeared, in part, to exert its influence through lifestyle and dietary habits although dietary information was not included in the study [18]. Low levels of education may associate with not only an unhealthy diet, but also lack of consistent and meaningful physical activity and increased alcohol consumption. Secondly, it is possible that increased educational attainment is associated with an increased awareness of the mechanisms of good health and wellness and blood pressure and cardiovascular maintenance. Lastly, high levels of education may be most influential on health outcomes, including hypertension, through occupational choices [18]. Among more than 150,000 study participants from South Africa, education was found to be inversely associated with hypertension for each race and sex group [19]. This inverse association remained when age was taken into account, was more striking in the younger age groups and in blacks, but was diminished in the highest weight classes [19]. Educational differences, however, do not fully account for the observed black-white differences in hypertension prevalence. Even at the higher education levels, the adjusted prevalence of hypertension remained nearly twice as high in blacks as in whites [19].

In the U.S. Family Blood Pressure Program Study, although education was inversely associated with blood pressure in the total population, within-group analyses showed that education remained a significant predictor of blood pressure only among African Americans [3]. Racial disparities in blood pressure may be better explained by differences in education than by genetic ancestry. Similarly, even though some genetic variants have been found, the full genetic landscape of type 2 diabetes, especially among African-Americans, is not yet discovered [20]. Therefore, studies assessing the relationships between education and hypertension/diabetes should provide practical targets for addressing these health disparities. Our results showing large differences among black individuals in the U.S., but little difference in Jamaica raise important questions, with regards to the effect of social context on health risks.

The important difference between the two countries of the association between education and hypertension/diabetes might lie in the large difference between the obesity prevalence of the two samples. Obesity trends that show rampant increase in obesity are likely to increase social disparities in diabetes and hypertension [21]. The magnitude of this effect depends on the strength of the relationship between obesity and diabetes/hypertension across categories of disadvantage. Therefore, blacks and non-Hispanic whites in the United States are expected to have a higher health disparity for diabetes and hypertension. Thus contrasting black populations within the United States and Caribbean countries will be informative for ways to diminish the health disparities related to education. It has been shown that, in African Americans, the relationship between inflammatory markers and insulin resistance/diabetes is mediated by adiposity [22]. Foreign-born blacks in the U.S. generally had lower obesity levels compared to their U.S.-born counterparts, which cannot be explained by socioeconomic status (SES) only [23].

It is interesting to comment that although in actual-dollar values the income of the JHS participants is higher than that of the Jamaican counterparts, other discriminatory phenomena, such as psychosocial stress and racism, in the U.S. context, but less common in the Caribbean might play a role. Additionally, both samples are mainly urban, minimizing the influence of the urban-rural influences on the two outcomes. Consistent rural-to-urban and socioeconomic gradients were found in prevalence of elevated blood pressure among adolescents in Poland, which increased with continuous lines from large cities through small- to medium-sized cities to villages and from high-SES to low-SES familial environments [24]. The adjusted likelihood of developing systolic and diastolic hypertension decreased with each step increase in maternal educational attainment and increased urbanization category [24].

Studies have shown that, among SES indicators, it is education and not income that is related to incidence and prevalence of disease [25]. The principal factor acting to reduce increases in diabetes prevalence is increases in education which by itself predicted a significant decline in diabetes prevalence [26]. Along with studies that assessed the relationship between SES and diabetes care [27–29], our investigation that assessed the relationship between diabetes and education among black individuals is very timely and relevant. Large, recent migration patterns from the non-Hispanic Caribbean islands and Africa have increased the share of U.S. blacks born outside of the United States. Still little is known about health patterns in these foreign-born populations. Our project report adds insights into the different stages of epidemiological transition that African-descent individuals in both settings are involved in (namely, the shift in cause-of-death patterns that comes with the overall decline of death rates), and add information to the much debated ‘thrifty gene hypothesis’ that has so many applications to cardiometabolic diseases such as hypertension and diabetes. The fundamental basis of the ‘thrifty gene hypothesis’ (proposed more than half-a-century ago) is that, in our early evolutionary history, genes that promoted efficient fat deposition would have been advantageous because they allowed their holders to survive at periods of famine. In modern society, such genes are disadvantageous because they promote fat deposition in preparation for a famine that never comes, and the result is widespread obesity and diabetes. These arguments have been criticized recently [30]. It appears that measures of socioeconomic status such as education is a main player in the overall transition, and thus are important factors to consider when addressing the genetic components.

Among the limitations of our investigation is the fact that diet and physical activity levels could be different in Jackson, MS and Spanish Town, Jamaica, as our assessed sites have very different cultures. Unfortunately, whereas both cohorts attempted to collect information on diet and physical activity, the data protocols and methodologies used to collect these variables were very different in the individual studies, thus making comparison across the two sites not feasible. For example, within the JHS a specifically adapted ‘Deep South’ food frequency questionnaire was used (derived from the FFQ used in the US Atherosclerosis Risk in Communities cohort), very difficult to extrapolate to the food intake questionnaire used in STCS. Similarly, a very different methodology was used to asses basic physical activity level in STCS, as opposed to the 24-points scale used to assess the four components of physical activity in the JHS.

## Conclusions

Studies have suggested that social inequalities in chronic disease outcomes differ between industrialized and developing countries, but few have directly compared these effects. There were differences in the patterns of educational health disparity for hypertension and diabetes mellitus comparing African-descent individuals in Jamaica and the USA. In Jamaica, educational disparities were largely explained by age, sex and BMI, while in the USA these disparities were larger and persisted after accounting these variables.

## Additional file

Additional file 1:
**Table S1.** Sex-Specific Characteristics of Study Participants in Spanish Town Cohort Study and Jackson Heart Study. **Table S2.** Sex Differences in the Characteristics of Study Participants in Spanish Town Cohort Study and Jackson Heart Study. **Figure S1.** Distribution of Education Level by Age Category (A) Spanish Town (B) Jackson Heart Study. **Figure S2A.** Prevalence of hypertension by age category in Spanish Town Cohort and Jackson Heart Study. **Figure S2B.** Prevalence of diabetes by age category in Spanish Town Cohort and Jackson Heart Study. (DOCX 67 kb)

## Abbreviations

BMI: Body mass index
HS: High school
JHS: Jackson Heart study
PR: Prevalence ratio
SES: Socioeconomic status
STC: Spanish Town Cohort Study
