# Supplementary material for: Educational health disparities in hypertension and diabetes mellitus among African descent populations in the Caribbean and the USA: a comparative analysis from the Spanish town cohort (Jamaica) and the Jackson heart study (USA)
Source: Int J Equity Health. 2017 Feb 14;16:33. doi: 10.1186/s12939-017-0527-9 (PMC5320798; doi:10.1186/s12939-017-0527-9)
Supplement: Additional file 1: — Table S1. Sex-Specific Characteristics of Study Participants in Spanish Town Cohort Study and Jackson Heart Study. Table S2. Sex Differences in the Characteristics of Study Participants in Spanish Town Cohort Study and Jackson Heart Study. Figure S1. Distribution of Education Level by Age Category (A) Spanish Town (B) Jackson Heart Study. Figure S2A. Prevalence of hypertension by age category in Spanish Town Cohort and Jackson Heart Study. Figure S2B. Prevalence of diabetes by age category in Spanish Town Cohort and Jackson Heart Study. (DOCX 67 kb) [file 12939_2017_527_MOESM1_ESM.docx]

**Supplementary Table 1: Sex-Specific Characteristics of Study Participants in Spanish Town Cohort Study and Jackson Heart Study**

| Characteristic | Spanish Town Cohort | Jackson Heart Study | p-value |
| --- | --- | --- | --- |
| MEN | N = 944 | 1787 |  |
|  | Mean (SD) | Mean (SD) |  |
| Age (years) | 47.2 (14.3) | 53.1 (11.4) | <0.001 |
| Height (cm) | 172.3 (7.2) | 177.7 (6.8) | <0.001 |
| Weight (kg) | 71.6 (14.1) | 94.8 (21.2) | <0.001 |
| Body Mass Index (kg/m^2^) | 24.1 (4.3) | 30.0 (6.1) | <0.001 |
| Systolic Blood Pressure (mmHg) | 121.5 (20.4) | 127.7 (17.4) | <0.001 |
| Diastolic Blood Pressure (mmHg) | 70.0 (14.3) | 81.9 (10.5) | <0.001 |
| Fasting Plasma Glucose (mmol/l) | 5.47 (2.20) | 5.54 (1.78) | 0.321 |
|  | % (n) | % (n) |  |
| Hypertension | 22.4 (211) | 59.5 (1063) | <0.001 |
| Diabetes | 10.4 (98) | 16.4 (293) | <0.001 |
| Education Category |  |  | <0.001 |
| *Less than High School* | 61.9 (584) | 16.8 (301) |  |
| *High School* | 24.9 (235) | 19.7 (352) |  |
| *More than High School* | 13.2 (125) | 63.5 (1134) |  |
|  |  |  |  |
| WOMEN | N = 1438 | N = 3079 |  |
|  | Mean (SD) | Mean (SD) |  |
| Age (years) | 46.1 (13.4) | 54.2 (11.2) | <0.001 |
| Height (cm) | 161.0 (6.4) | 164.1 (6.3) | <0.001 |
| Weight (kg) | 73.2 (17.1) | 89.1 (21.1) | <0.001 |
| Body Mass Index (kg/m^2^) | 28.2 (6.4) | 33.1 (7.6) | <0.001 |
| Systolic Blood Pressure (mmHg) | 120.2 (21.5) | 126.0 (18.0) | <0.001 |
| Diastolic Blood Pressure (mmHg) | 69.1 (13.6) | 77.6 (10.1) | <0.001 |
| Fasting Plasma Glucose (mmol/l) | 5.57 (2.33) | 5.51 (1.83) | 0.375 |
|  | % (n) | % (n) |  |
| Hypertension | 26.7 (384) | 63.7 (1962) | <0.001 |
| Diabetes | 13.8 (199) | 19.3 (593) | <0.001 |
| Education Category |  |  | <0.001 |
| *Less than High School* | 61.6 (886) | 16.1 (495) |  |
| *High School* | 23.6 (339) | 20.6 (634) |  |
| *More than High School* | 14.8 (213) | 63.3 (1950) |  |

**Supplementary Table 2: Sex Differences in the Characteristics of Study Participants in Spanish Town Cohort Study and Jackson Heart Study**

| Characteristic | Males | Females | p-value |
| --- | --- | --- | --- |
| SPANISH TOWN COHORT | N = 944 | 1438 |  |
|  | Mean (SD) | Mean (SD) |  |
| Age (years) | 47.2 (14.3) | 46.1 (13.4) | 0.081 |
| Height (cm) | 172.3 (7.2) | 161.0 (6.4) | <0.001 |
| Weight (kg) | 71.6 (14.1) | 73.2 (17.1) | 0.016 |
| Body Mass Index (kg/m^2^) | 24.1 (4.3) | 28.2 (6.4) | <0.001 |
| Systolic Blood Pressure (mmHg) | 121.5 (20.4) | 120.2 (21.5) | 0.134 |
| Diastolic Blood Pressure (mmHg) | 70.0 (14.3) | 69.1 (13.6) | 0.118 |
| Fasting Plasma Glucose (mmol/l) | 5.47 (2.20) | 5.57 (2.33) | 0.282 |
|  | % (n) | % (n) |  |
| Hypertension | 22.4 (211) | 26.7 (384) | 0.017 |
| Diabetes | 10.4 (98) | 13.8 (199) | 0.013 |
| Education Category |  |  | 0.493 |
| *Less than High School* | 61.9 (584) | 61.6 (886) |  |
| *High School* | 24.9 (235) | 23.6 (339) |  |
| *More than High School* | 13.2 (125) | 14.8 (213) |  |
|  |  |  |  |
| JACKSON HEART STUDY | N = 1787 | N = 3079 |  |
|  | Mean (SD) | Mean (SD) |  |
| Age (years) | 53.1 (11.4) | 54.2 (11.2) | 0.085 |
| Height (cm) | 177.7 (6.8) | 164.1 (6.3) | <0.001 |
| Weight (kg) | 94.8 (21.2) | 89.1 (21.1) | <0.001 |
| Body Mass Index (kg/m^2^) | 30.0 (6.1) | 33.1 (7.6) | <0.001 |
| Systolic Blood Pressure (mmHg) | 127.7 (17.4) | 126.0 (18.0) | 0.001 |
| Diastolic Blood Pressure (mmHg) | 81.9 (10.5) | 77.6 (10.1) | <0.001 |
| Fasting Plasma Glucose (mmol/l) | 5.54 (1.78) | 5.51 (1.83) | 0.245 |
|  | % (n) | % (n) |  |
| Hypertension | 59.5 (1063) | 63.7 (1962) | 0.005 |
| Diabetes | 16.4 (293) | 19.3 (593) | 0.017 |
| Education Category |  |  | 0.638 |
| *Less than High School* | 16.8 (301) | 16.1 (495) |  |
| *High School* | 19.7 (352) | 20.6 (634) |  |
| *More than High School* | 63.5 (1134) | 63.3 (1950) |  |

**Supplementary Figure 1: Distribution of Education Level by Age Category (A) Spanish Town (B) Jackson Heart Study**

*Panel A: Spanish Town Cohort*

HS = High School; p <0.001 for variation in education by age group

*Panel B: Jackson Heart Study*

HS = High School; p <0.001 for variation in education by age group; p-value accounts for clustering within families for Jackson Heart Study

**Supplementary Figure 2 A: Prevalence of hypertension by age category in Spanish Town Cohort and Jackson Heart Study**

P<0.001 for age difference in hypertension prevalence by age-category for both study sites

**Supplementary Figure 2 B: Prevalence of diabetes by age category in Spanish Town Cohort and Jackson Heart Study**

P<0.001 for age difference in diabetes prevalence by age-category for both study sites. P-value accounts for clustering of individuals within families in Jackson Heart Study
